# Uncommon association between vascular Ehlers–Danlos syndrome and ocular complications

**DOI:** 10.3389/fmed.2023.1089652

**Published:** 2023-03-29

**Authors:** Matei Popa Cherecheanu, Mihaela Oana Romanitan, Ruxandra Pirvulescu, Raluca Iancu, Gerhard Garhöfer, George Iancu, Alina Popa Cherecheanu, Mihail Zemba, Victor Vasile, Andrei Simonov, Daniel Branisteanu

**Affiliations:** ^1^Carol Davila University of Medicine and Pharmacy, Bucharest, Romania; ^2^Cardiovascular Surgery Clinic, Prof. Dr. Agrippa Ionescu Emergency Clinical Hospital, Bucharest, Romania; ^3^Department for Emergency Internal Medicine and Neurology, Stockholm South General Hospital, Stockholm, Sweden; ^4^Department of Ophthalmology, University Emergency Hospital, Bucharest, Romania; ^5^Department of Clinical Pharmacology, Medical University of Vienna, Vienna, Austria; ^6^Filantropia Clinical Hospital, Bucharest, Romania; ^7^Department of Ophthalmology, Clinical Military Emergency Hospital, Bucharest, Romania; ^8^Department of Interventional Radiology, University Emergency Hospital, Bucharest, Romania; ^9^Department of Ophthalmology, University of Medicine and Pharmacy Grigore T. Popa Iasi, Iasi, Romania

**Keywords:** vascular Ehlers–Danlos syndrome, vascular dissections, retinal artery occlusion, arterio-venous fistula, young persons

## Abstract

Ehlers–Danlos syndromes (EDS) represent a group of rare inherited disorders that affect connective tissues. There are 13 types of disease, most of them affecting joints or skin; symptoms usually include loose joints, joint pain, stretchy velvety skin, abnormal scar formation. However, the most serious type of disease is vascular EDS (vEDS), or EDS type 4 because patients may suffer vessels dissections or internal organs lesions, followed by bleeding, which endangers patient’s life, but also thromboembolic events. We present two clinical cases of vEDS managed in our clinic in 1 year distance. In both cases, patients were active young persons (in their thirties, and respectively, twenties), both with multiple non-traumatic vascular dissections, and severe ocular complications: arterio-venous fistula with massive exophthalmia, and central retinal artery occlusion, respectively. Both cases were challenging since the life of the patients were threatened by their condition. However, in both cases, prompt treatment and finding the right trigger of the ocular pathology and vascular injuries helped doctors to provide proper and prompt medical care, in order to prevent future similar events to happen and to preserve a good quality of life for these patients.

## Introduction

Ehlers–Danlos Syndromes (EDS) are a group of heritable connective tissue disorders, that are usually characterized by skin hyper elasticity, joint hyperlaxity and tissue fragility (1, 2). These syndromes appear due to abnormalities in biosynthesis and/or structure extracellular matrix proteins (3). Most types of EDS that have a known genetic cause, resulting from pathogenic variants of the genes that encode fibrillar collagen types I, III, and V, modifying or transforming enzymes of these collagens, or enzymes that play a crucial role in the biosynthesis of glycosaminoglycans (GAG) proteoglycan chains (4). The prevalence of EDS is estimated at 1 in 5000, without any ethnic or sex predilection (1, 2).

These syndromes were first identified in the early twentieth century, by dermatologists Edvard Ehlers and HenriAlexandre Danlos. They described patients with joint hypermobility, excessive skin extensibility, easy bruising and abnormal scar formation after injury (4). Even though patients with similar clinical aspects were previously reported by Russian and Danish dermatologists, Ehlers–Danlos disease had a chaotic history which delayed its identification (5).

Several classification structures have been proposed, but the most recent one was determined during the Ehlers–Danlos Syndrome International Symposium in 2017 (6), and includes 13 subtypes of EDS, divided into 7 pathogenic groups (2, 6). The reason it is so important to distinguish the different types of EDS, is because they do not develop in time the same complications and their prognosis is therefore different (6). Classic EDS (former types I and II) and especially hyper-mobile EDS (former type III) represent almost 85% of patients referred for consultation and vascular EDS type (type IV) about 5%. (1). The other EDS represent a small group of Mendelian genetic disorders whose clinical manifestation and causative genes are increasingly better characterized thanks to advances in genetics (1).

Vascular EDS (vEDS) is one if not the most challenging type of EDS. It is caused by mutations in the *COL3A1* gene (rarely *COL1A1*) (7). Patients often have a suggestive morphotype and thin skin revealing the subcutaneous venous network. They are often exposed to life-threatening complications: arterial complications (dissection, aneurysm, or arteriovenous fistula), spontaneous colonic perforations, pneumothorax and, in women’s case, risk of fragility of the uterine wall (particularly during childbirth) (6). Among all the medical problems to which patients with vEDS are exposed, vascular and/or organ rupture are the two most feared and deadly complications (8–10).

Beside the life-threatening complications, vEDS may also include ophthalmic findings such as: high refractive errors (e.g., myopia – 25.3%), irregular astigmatism, steep keratometry values, convergence insufficiency, strabismus, thin pachymetry, increased applanation-related stromal folds. Among the symptoms the patients experience, the most upsetting are blurred vision, difficulty focusing at near, binocular diplopia, dry eye sensation, headaches (2, 10).

Eye examination of EDS patients may identify ptosis (32%), infraorbital creases (29.3%), epicanthal folds (18.6%), hypertelorism (8%), strabismus (8%), blue sclerae, lenticular changes, corneal thinning (11).

However, besides minor ophthalmic signs and symptoms, sometimes major events with direct or indirect ophthalmic implications may occur. Among those, there are retinal artery occlusions or carotid-cavernous fistula (11).

Vascular Ehlers–Danlos syndrome (vEDS) should be considered in individuals with any one of the major diagnostic criteria or several minor diagnostic criteria, particularly in those under 40 years (see Table 1) (11).

**Table 1:** Major and minor diagnostic criteria for vEDS (11).

Major diagnostic criteria	Minor diagnostic criteria
•Arterial aneurysms, dissection, or rupture	•Thin, translucent skin (especially noticeable on the chest/abdomen)
•Intestinal rupture	•Characteristic facial appearance (narrow nose, thin lips, micrognathia, prominent eyes)
•Uterine rupture during pregnancy	•Acrogeria (an aged appearance to the extremities, particularly the hands)
•Family history of vEDS	•Carotid-cavernous sinus arteriovenous fistula
	•Hypermobility of small joints
	•Tendon/muscle rupture
	•Early-onset varicose veins
	•Hemo/pneumothorax
	•Easy bruising (spontaneous or with minimal trauma)
	•Chronic joint subluxations/dislocations
	•Congenital dislocation of the hips
	•Clubfoot

Clinical diagnostic criteria established in 2017 the opportunity of genetic testing. The certainty diagnosis of vEDS is determined either by identification of a heterozygous pathogenic variant in *COL3A1* on molecular genetic testing, by abnormalities in synthesis and mobility of type III collagen chains on biochemical analysis of type III procollagen from cultured fibroblasts, or even by histopathological examination of tissue biopsy; the latter one may be challenging, since histopathological findings of vEDS are not widely recognized (12).

Studies show that the majority (60%) of individuals with vEDS diagnosed under age of 18 are identified because of a positive family history (13). Death usually occurs in the first two decades of life almost always because of spontaneous artery rupture or dissection. Artery rupture, 60% of which involved the aorta, was responsible for all deaths in young males. Death before age 20 years was seen in a 3:1 ratio of males: females. In adults with a *COL3A1* pathogenic variant, vascular rupture or dissection and gastrointestinal perforation or organ rupture are the presenting signs in 70% of cases (4).

Complications in vEDS patients are dramatic and often unexpected, presenting as sudden death, stroke and its neurologic sequelae, acute abdomen, retroperitoneal bleeding, uterine rupture at pregnancy, and/or shock. Vascular complications include rupture, aneurysm, and/or dissection of major or minor arteries. Arterial rupture may be preceded by aneurysm, arteriovenous fistulae, or dissection, or may occur spontaneously. The sites of arterial rupture are the thorax and abdomen (66%), head and neck (17%), and extremities (17%). Patients may also experience hemothorax and hemopneumothorax, often in association with pulmonary blebs, cystic lesions, and hemorrhagic or fibrous nodules, often followed by hemoptysis can be severe and recurrent, even life threatening (14, 15).

We hereby present two clinical cases of vEDS, each one presented in ER with different severity, one of them with both general life threatening and sight-threatening conditions and the other one with a sudden decrease in visual acuity, without other apparent signs and symptoms. In this latter case, further tests revealed that beyond clinical signs, may lie dangerous disorders and proper care and treatment made the difference between life and death.

## Case 1

### Carotid cavernous fistula in vEDS

A 28-year-old female patient presented to the ER for binocular diplopia, right axial proptosis, right hemicrania, tinnitus, pronounced vertigo when changing position. The disorders lasted for 2–3 days and were accompanied by nausea and vomiting, for approximately 24 h before the time of admission. The patient did not have any knowledge of an underlying medical condition, such as EDS.

During hospitalization, the patient complains of intense pain in the lumbar area and in the left flank with sudden onset and anterior irradiation. The general condition deteriorated, and the patient became hemodynamically unstable with 70/40 mmHg ABP, cardiac frequency 120/min, signs of hemorrhagic sock. The abdominal CT exam revealed an anatomic anomaly with two left renal arteries, one smaller 2 mm and the other 4 mm diameter, the latter with an aneurismal dilation (8/7 mm in one axis and 4/5 in the other) localized at 21 mm of the origin of the renal artery from the aorta, voluminous retroperitoneal, peripancreatic and peri-renal hematoma as well as left kidney infarction. Emergency surgical intervention was performed, which consisted in left nephrectomy and left adrenalectomy of necessity, restoration of aortic arterial flow by interposition of DACRON 18 prosthesis following aortic abdominal rupture (due to an aortic aneurism which was identified during surgery and explained the massive hematoma). Postoperative, the evolution was difficult with acute kidney failure, need of renal replacement therapy (veno-venous hemodiafiltration CCVHD) because of hyperkalemia and metabolic acidosis.

When the patient was hemodynamically stabile, she was transferred to the Ophthalmology Department of the Emergency Hospital of Bucharest for right carotid cavernous fistula suspicion. The presence of a lumbar suppurative wound at the nephrectomy incision was noted on his arrival at our clinic.

The visual acuity in the right eye was approximately 1/50 without correction. The examination was performed with the patient lying horizontally, due to the recent surgery, so a best corrected visual acuity was not possible. Intraocular pressure was elevated in the right eye (24 mm Hg) and within normal limits in the left eye (12 mm Hg), measured by Perkins applanation tonometer.

**Figure 1:**
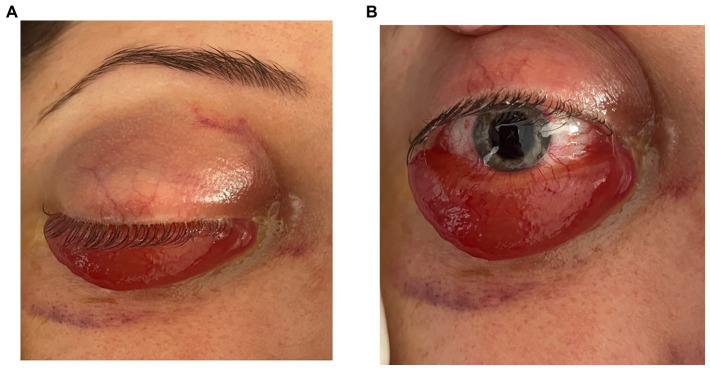
**(A,B)** Frontal view showing axial proptosis, as well the massive chemosis with conjunctival prolapse over the lower eyelid.

Clinical examination in the right eye revealed marked conjunctival hyperemia with massive conjunctival chemosis and prolapse of the conjunctiva over the lower lid, axial proptosis, and internal and external ophthalmoplegia (Figures 1A,B). Cortico-nuclear lens opacities were identified in both eyes. Fundus exam of both eyes showed slightly tortuous vessels, more obvious in the right eye than in the left eye.

**Figure 2:**
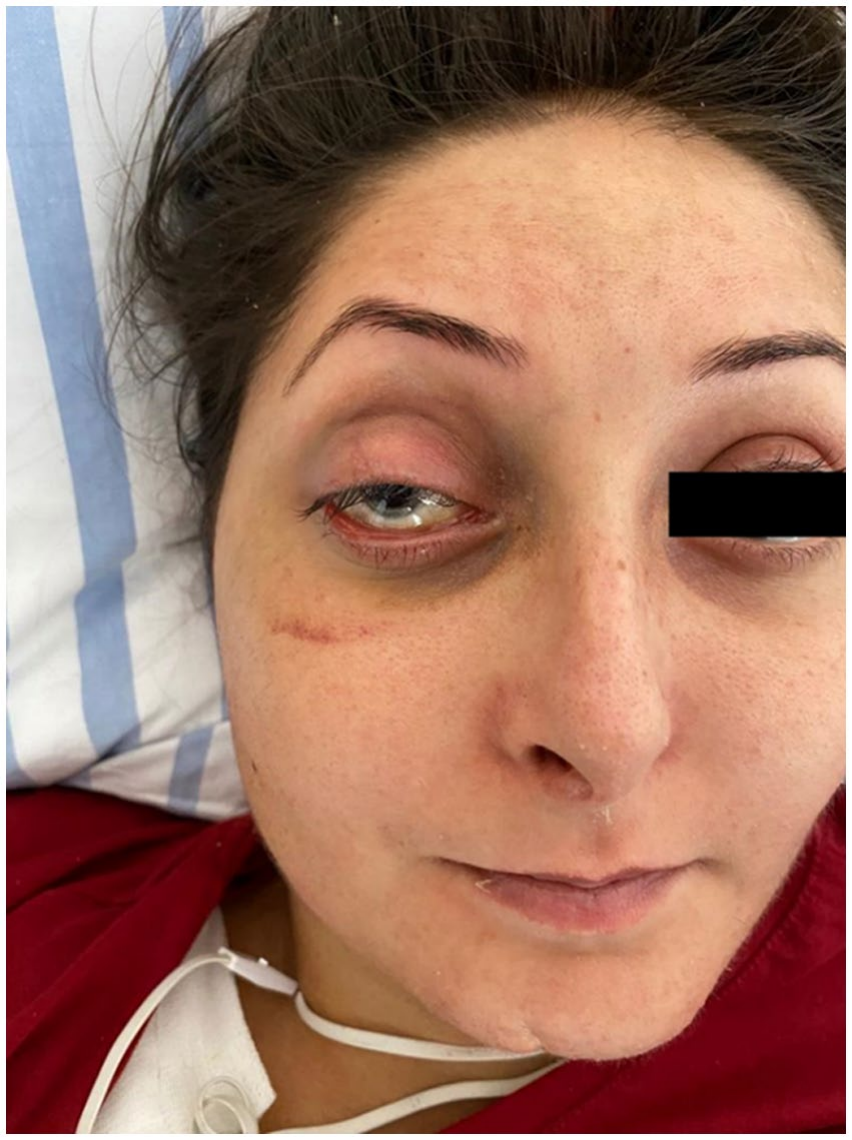
Patient’s appearance: narrow nose, translucent skin, thin vermilion lips.

Another aspect that caught our attention was the particular appearance of the patient: narrow nose, translucent skin, thin vermilion lips (Figure 2).

**Figure 3:**
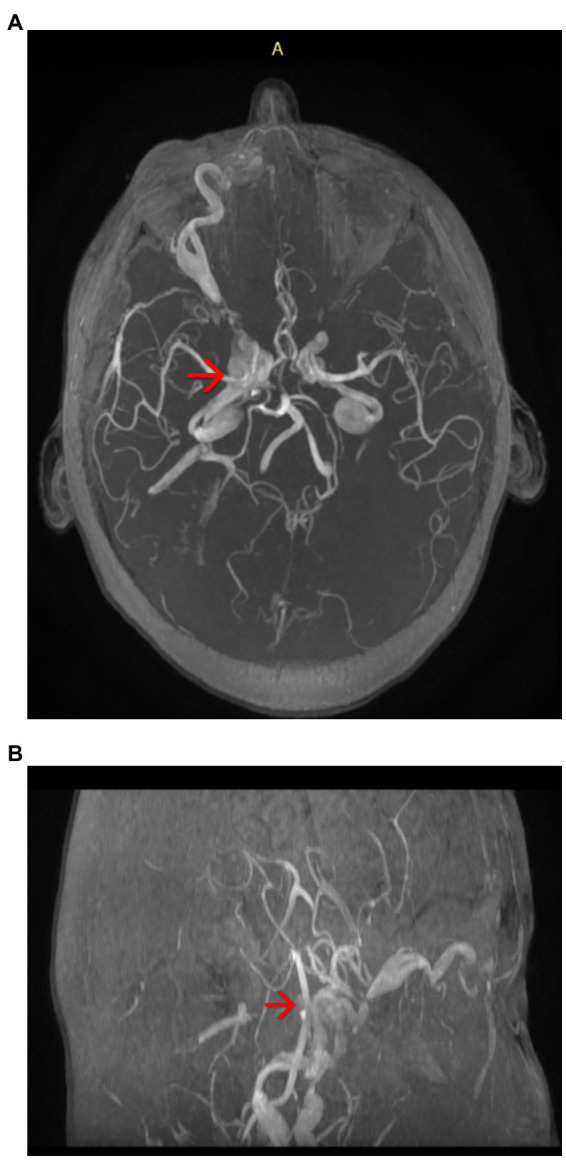
**(A,B)** Magnetic resonance angiography (MRA) of the brain highlighting the arterialization of the cavernous sinus (axial plane) – see red arrows.

**Figure 4:**
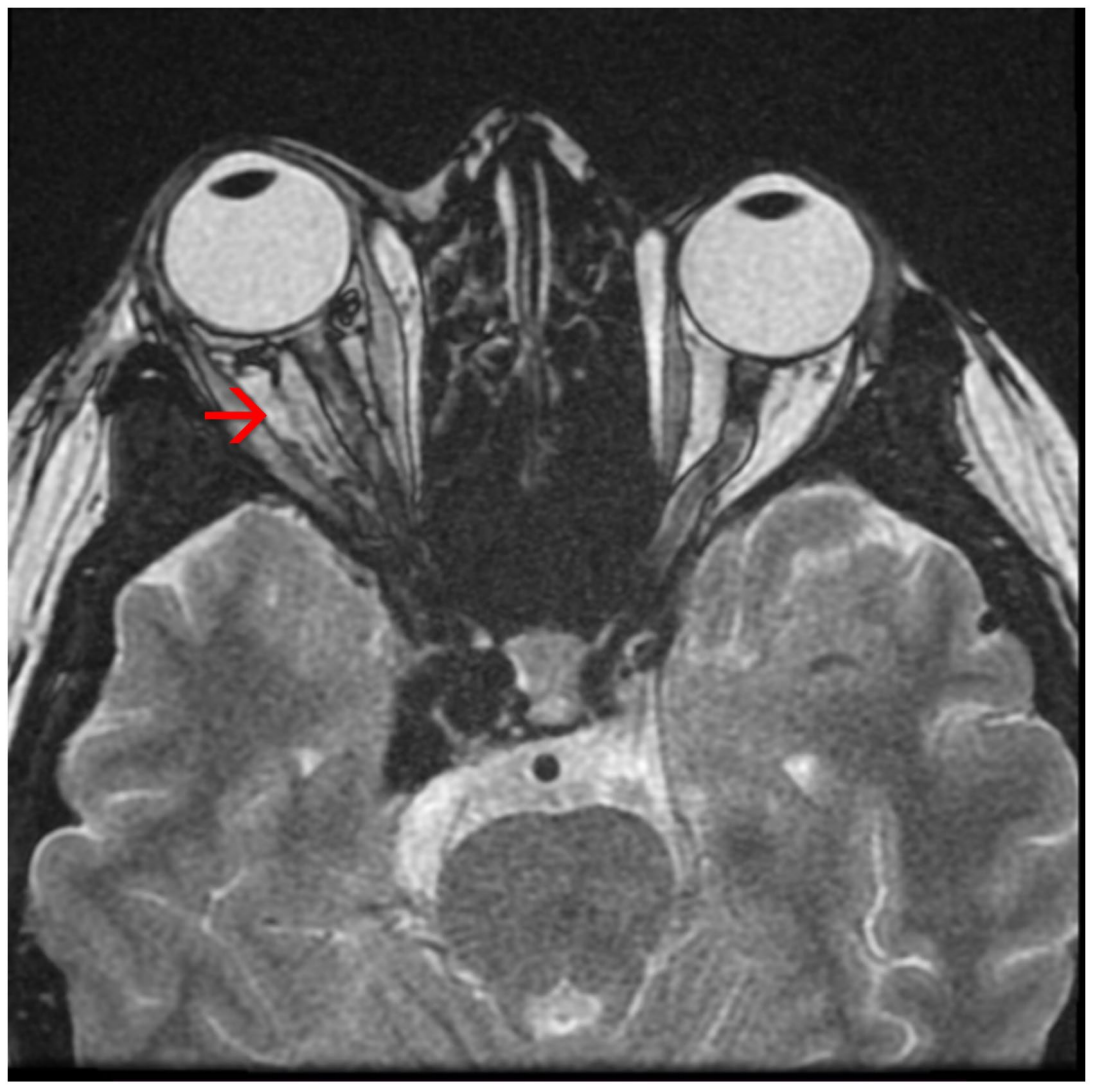
Magnetic resonance angiography (MRA) showing axial proptosis and dilation of the superior right ophthalmic vein – see red arrow.

The brain Magnetic Resonance Angiography (MRA) examination revealed:

- Right carotid-cavernous fistula (arterialization of the cavernous sinus) (Figure 3)
- Axial proptosis, dilatation of the superior right ophthalmic vein, oedema of the intraconal fat, enlarged eye muscles in diameter on the right side (Figure 4)
- Subacute stage meningeal hemorrhage in the right paratentorial area and left peri- and intergyral fronto-parietal area,
- Subacute hemorrhagic spot intraparenchymal cerebellar hemisphere on the left side,
- Pituitary adenoma,
- Right frontal sinusitis.

**Figure 5:**
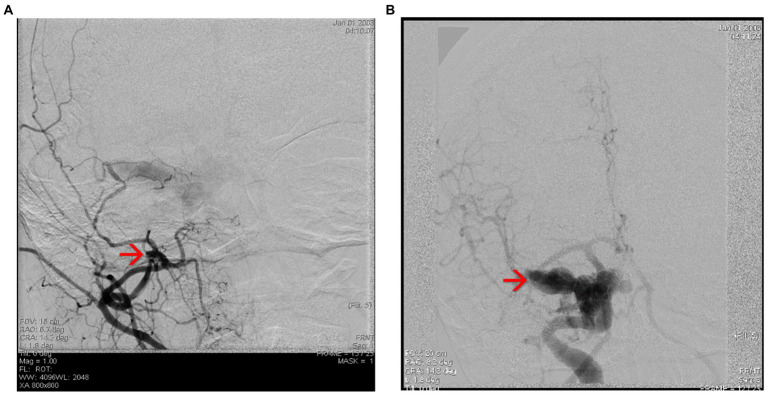
**(A,B)** Cerebral angiography highlighting the Type D carotid-cavernous fistula [according to Barrow classification (4)] on the right side, and dural shunts between both branches from the external carotid artery (ECA) ant the internal carotid artery (ICA).

**Figure 6:**
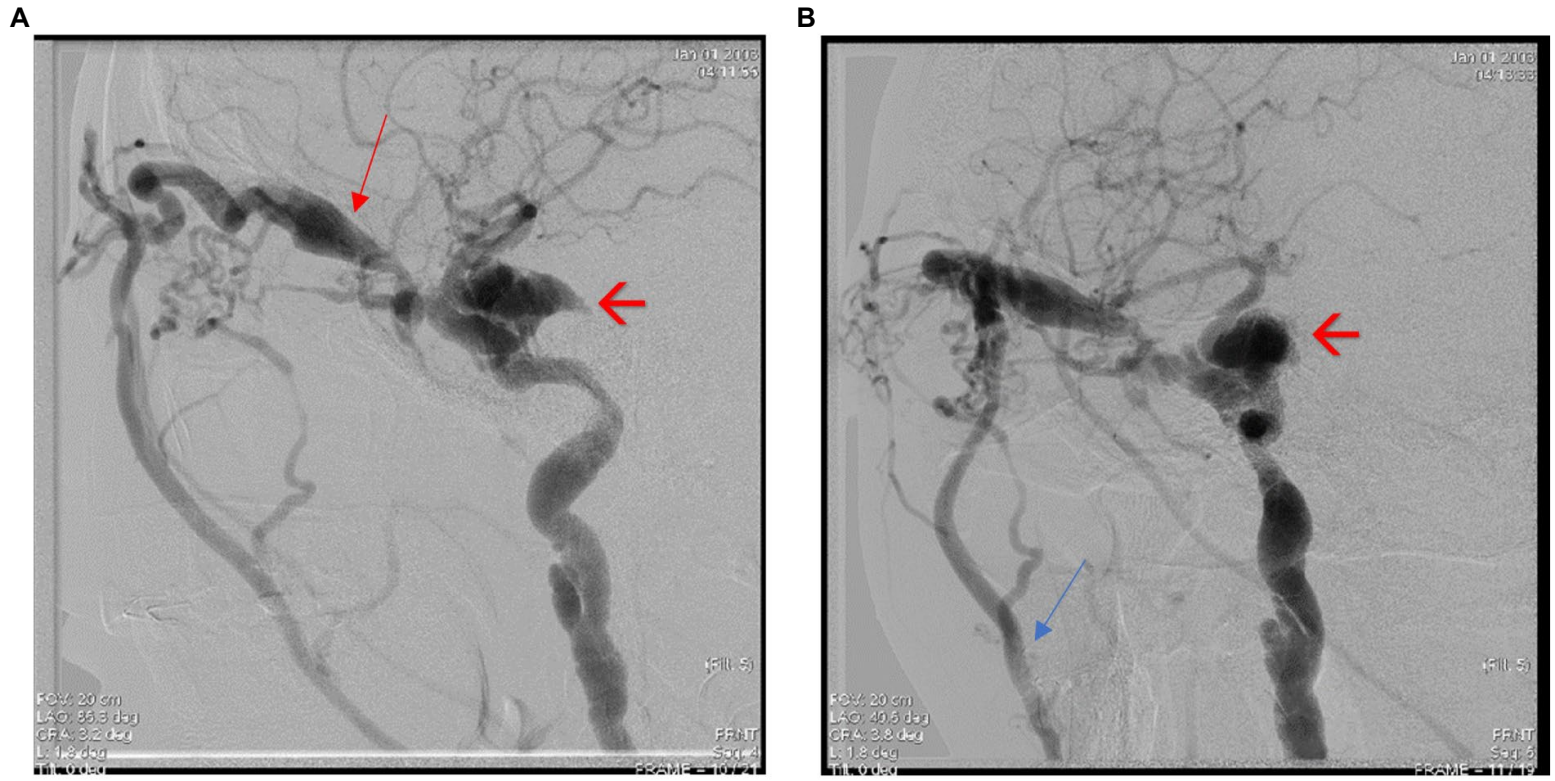
**(A,B)** Cerebral angiography highlighting the direct carotid-cavernous fistula (Type D). Notice the dilated superior ophthalmic vein – see red arrow, and the shunts between ECA and ICA and the facial vein – see blue arrow.

The patient was referred to the Interventional Neurology department for cerebral angiography and subsequent embolization of the carotid-cavernous fistula. Cerebral angiography revealed a direct carotid-cavernous fistula (Type D) with discharge through the right ophthalmic vein and subsequently at the level of both facial veins, as well as stenosis at the level of the M1 segment of the left middle cerebral artery (MCA) of approx. 80% with small post-stenotic dilations. Irregular appearance of the medium and small branches of the left anterior cerebral artery (ACA) and MCA can be observed (Figures 5A,B, 6A,B).

**Figure 7:**
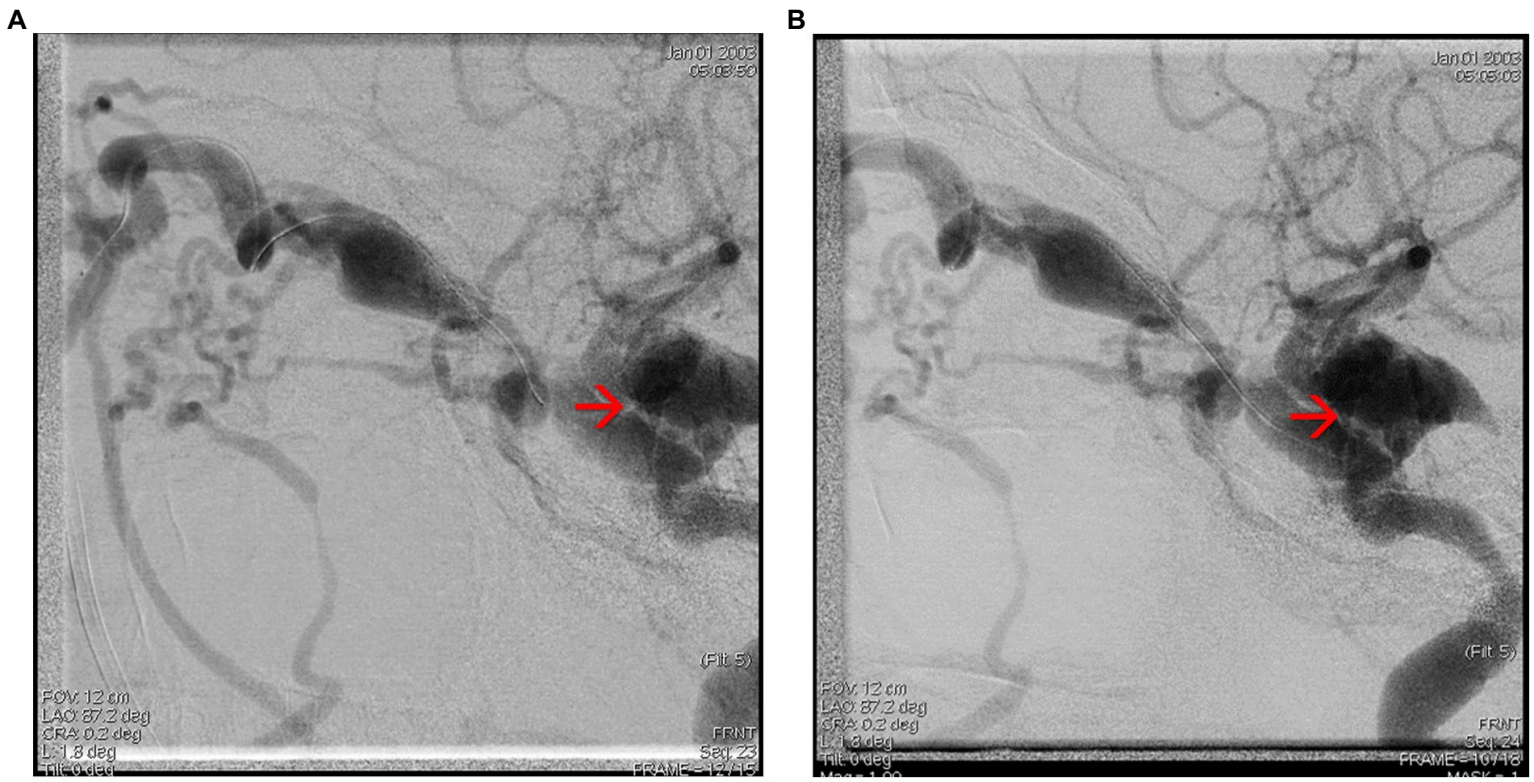
**(A,B)** Cerebral angiography capture showing embolization of the carotid-cavernous fistula through the facial vein – see red arrows.

**Figure 8:**
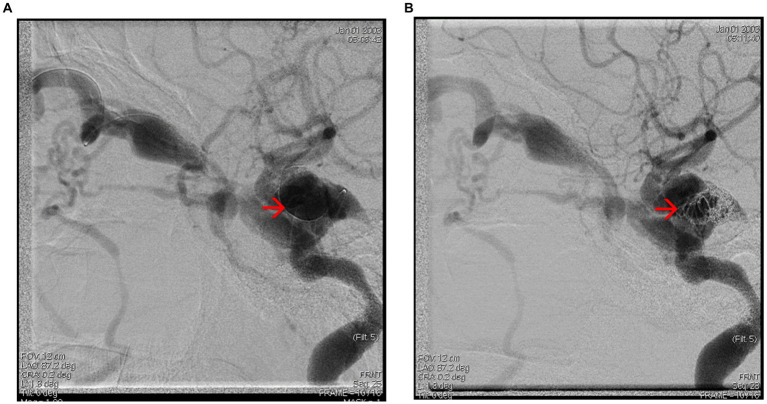
**(A,B)** Cerebral angiography captures showing embolization of the carotid-cavernous fistula – see red arrows.

**Figure 9:**
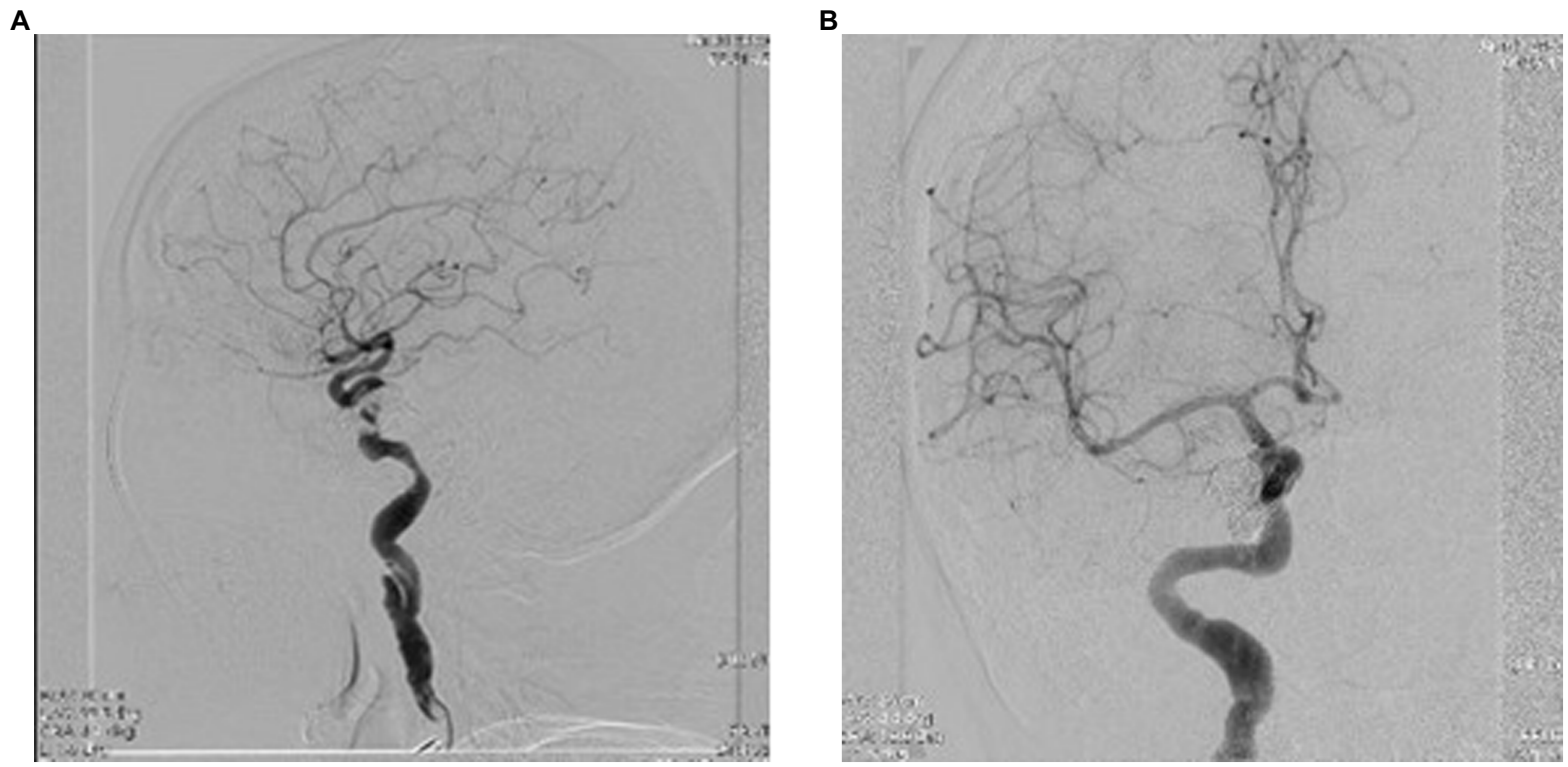
**(A,B)** Final ICA injection showing complete embolization of the carotid cavernous fistula, frontal, and lateral view.

Interventional treatment was performed by embolizing the carotid-cavernous fistula through a right femoral vein approach. Multiple embolization coils were deployed at the level of the right cavernous sinus *via* a right facial vein approach and an optimal post-embolization result was obtained with no loading at the level of the fistula, without any peri-procedural neurological incidents (Figures 7A,B, 8A,B). Figures 9A,B show the complete embolization of the carotid cavernous fistula.

**Figure 10:**
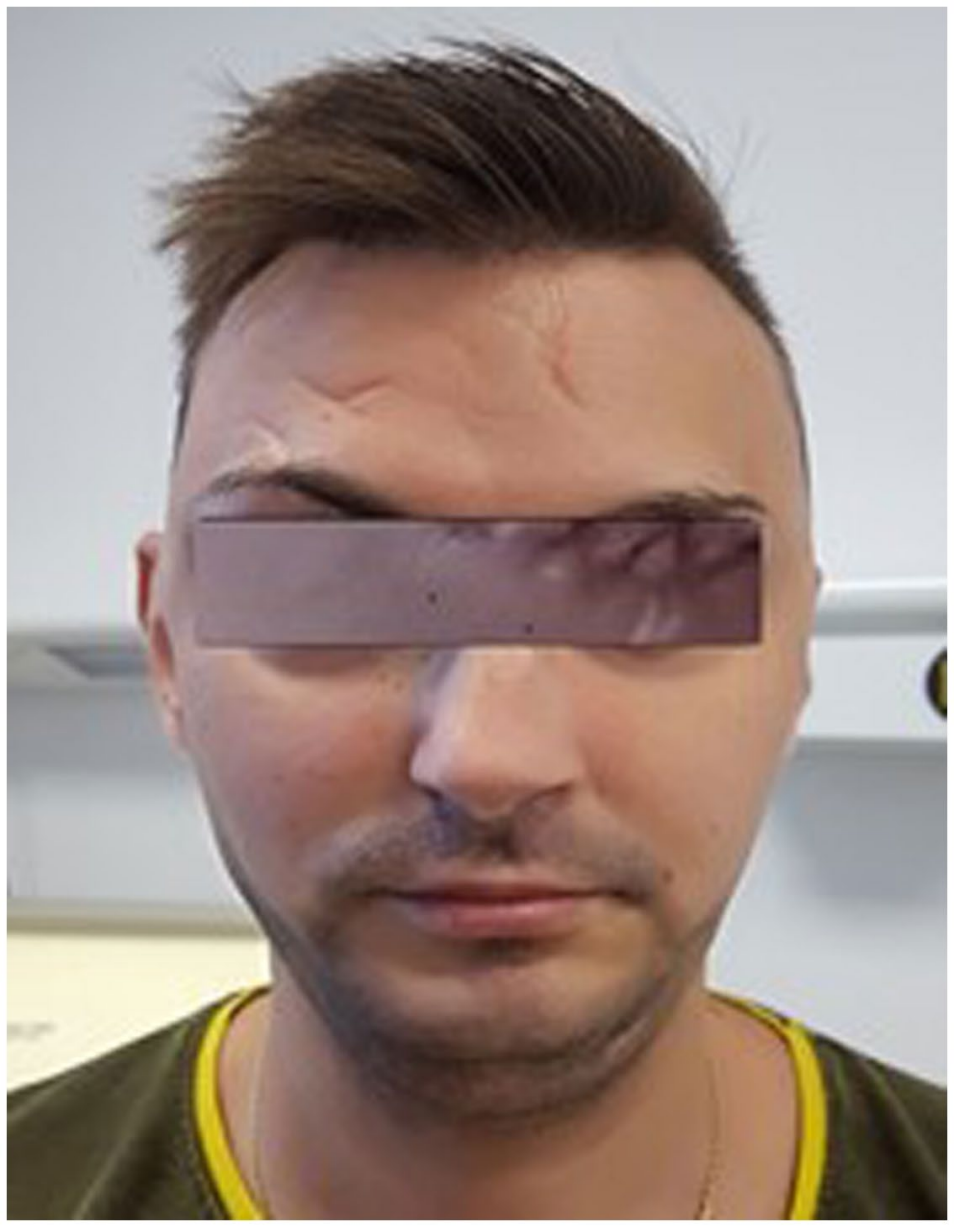
Front view of the patient, 1 month after the angiographic intervention.

One month after the intervention, remission of axial proptosis and chemosis, recovery of external and internal ocular motility can be observed (Figure 10).

Given the nature of the patient’s injuries and the clear fulfilment of several major and minor criteria for vEDS (multiple vascular lesions, the clinical general and ophthalmic aspects, and the physical appearance of the patient), we did not consider a skin biopsy as initial diagnosis test, instead, patient underwent specific genetic tests that confirmed the diagnosis of vascular Ehlers–Danlos syndrome, by finding missense mutation in exon 39 of the *COL3A1* gene.

## Case 2

### Central retinal artery occlusion (CRAO) in vEDS

**Figure 11:**
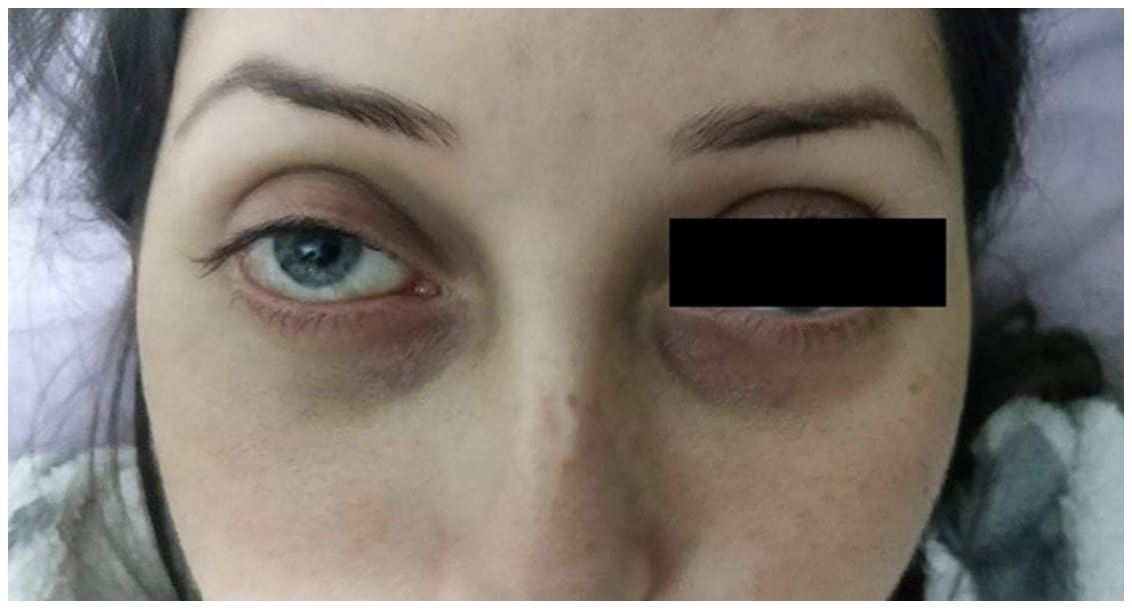
Appearance of the 28 years old patient presented in the ER with CRAO in the left eye.

The second case we present in this paper is a 28-year-old male, from the urban environment, who presented in the ER with sudden, painless decrease of visual acuity in the left eye, 2 weeks before. The general aspect of the patient was similar with the previous case: translucent skin, narrow nose, thin vermilion lips (Figure 11). From the medical history of the patient arouse: premature birth (2 months before term), loss of consciousness after a cranio-cerebral trauma (10 years *a priori*), capillary fragility with easy bruising, and patient’s father – known with ischemic heart disease (two stents). Same as the previous clinical case, the patient was not aware of any medical condition such as EDS.

**Figure 12:**
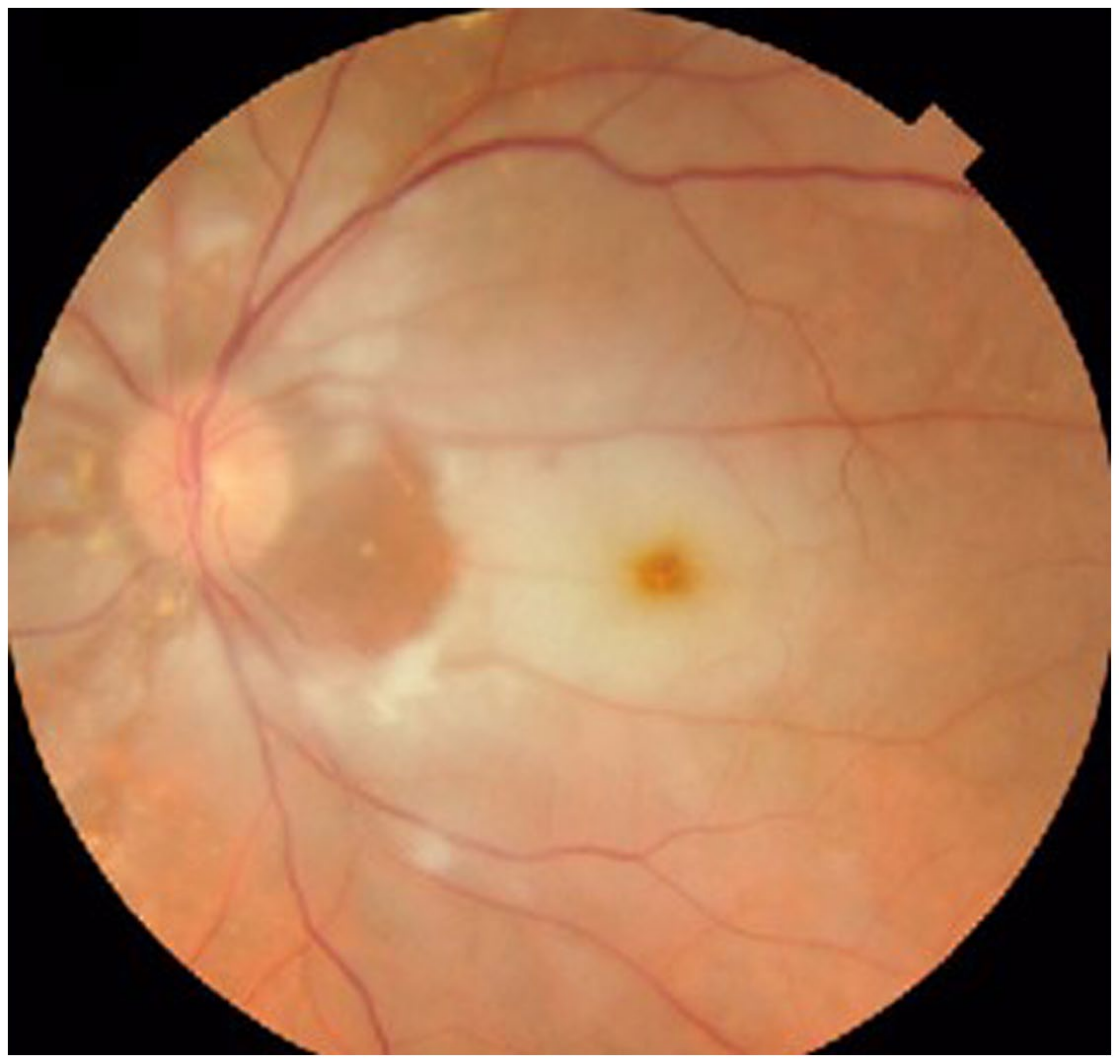
Aspect of the left eye fundus showing CRAO with cilioretinal artery sparing.

The ophthalmological exam showed best corrected visual acuity in the right eye 50/50, and no central vision in the left eye. Visual field was within normal limits in the right eye, while in the left eye only a small temporal island of vision was identified. Intraocular pressure was within normal limits in both eyes; IOP in both eyes was 15 mmHg. Eye fundus in the right eye showed slightly narrowed retinal vessels, while in the left eye it showed an optic disc with discrete blurred margins (superiorly and inferiorly), whitish retinal oedema, small, perfused area between the optic disc and the macula (patent cilioretinal artery), and thin retinal vessels (Figure 12).

The general clinical exam was within normal limits. Blood tests showed a slightly delayed prothrombin time (PT = 18 s) and an international normalized ratio (INR) of 1.65, which is considered a high value for a healthy person.

**Figure 13:**
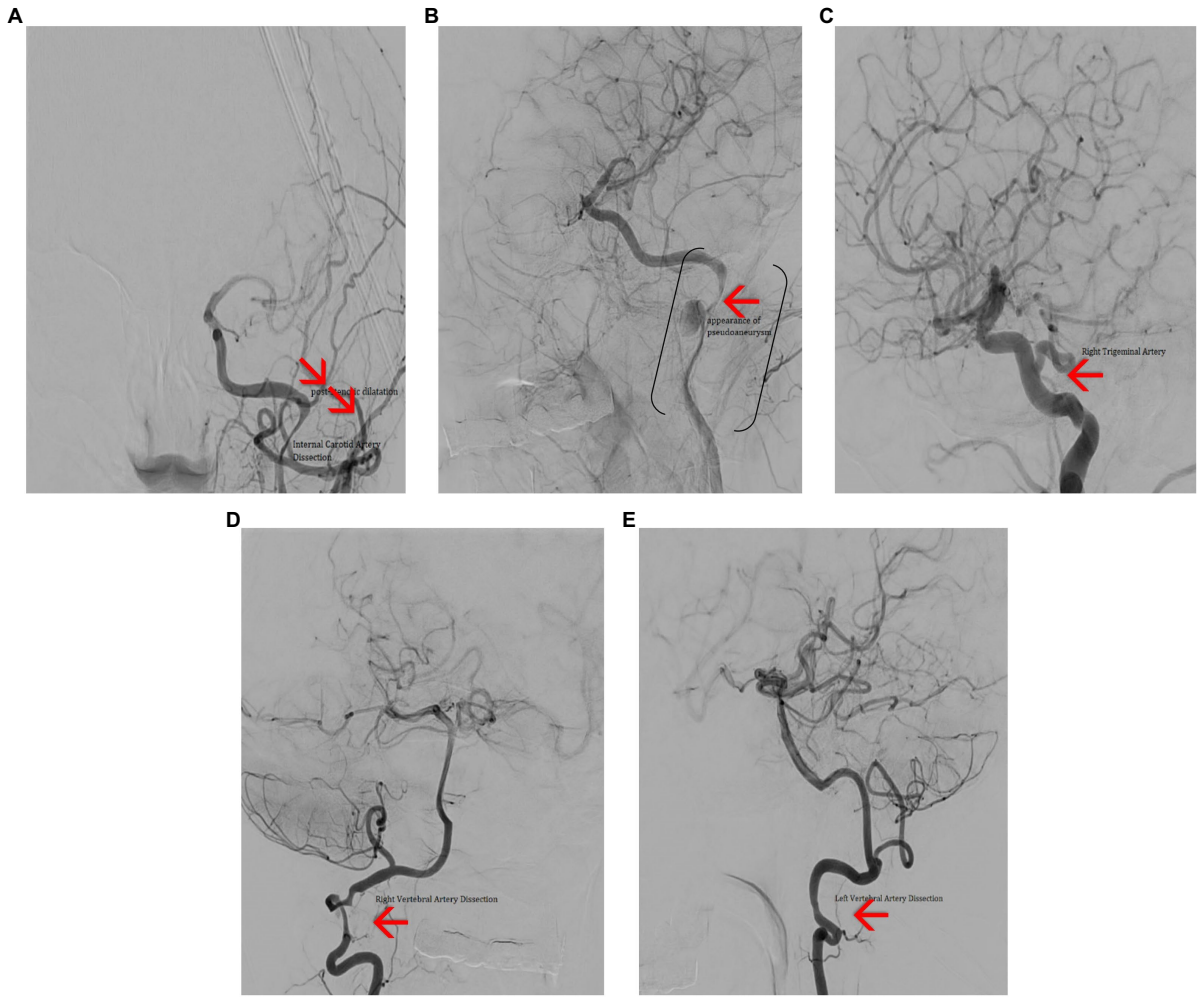
Cerebral angiography **(A)** – frontal view showing dissection in the C2–C3 segments of the left ICA, with post stenotic dilatation and poor intracranial filling. **(B)** Left anterior oblique view showing dissection of the C2–C3 segments of the ICA (brackets) and a pseudo-aneurism (red arrow). **(C)** Lateral view showing persistent right trigeminal artery (red arrow). **(D)** Frontal view showing right VA dissection (red arrow). **(E)** Lateral view showing left VA dissection (red arrow).

Further examinations included a transcranial echo-Doppler exam, which revealed decreased velocity flow in the left internal carotid artery (ICA). Following the neurological exam, cerebral angiography was indicated (Figures 13A–E), and it exposed:

- dissection at the left ICA level, image of a pseudoaneurysm adjacent to the dissection,
- significant hemodynamic stenosis
- suggestive aspects of dissection in both vertebral arteries
- an anatomical variant of the right trigeminal artery

Patient was referred to Neurology Clinic and underwent percutaneous transluminal angioplasty with left ICA stenting.

Given the fact that patient had a significant ophthalmic event, sub-clinical non-traumatic vascular dissections, history of capillary fragility with repeated ecchymosis, as well as an appearance with narrow nose, translucent and elastic skin and small stature, a skin biopsy was performed, revealing changes in the elastic fibers and fibrillar collagen suggestive for vEDS. More specifically, the tissue sections revealed knots of elastic fibers in transverse and oblique sections, with marked pathological changes. Some areas showed elastic fibers disintegrated almost completely, while other remnants elastic fibers had an annular, relatively round appearance and other elastic fibers had an irregular, wavy, garlanded, outline; the modifications were present in all analyzed skin samples, in variable size.

Subsequent genetic tests confirmed the diagnosis, by finding a mutation on *COL3A1* gene.

Patient underwent anticoagulant treatment, with favorable results, was advised to avoid any physical effort that might put him at risk for vascular dissections and continues to manage his condition properly and carefully.

## Discussion and conclusion

Vascular Ehlers–Danlos syndrome (vEDS) is a particular type of EDS characterized by multiple organ fragility, arterial, intestinal, and/or uterine; patient’s skin appears thin and translucent; among the general clinical aspects of the disease, are included easy bruising, characteristic facial appearance (thin vermilion of the lips, micrognathia, narrow nose, prominent eyes), which were present in both our patients’ case; extremities of these patients have an aged appearance particularly involving the hands (see Table 1). Individuals with vEDS are unfortunately inclined to vascular dissections or ruptures, gastrointestinal perforations, or even organ ruptures, as pointed out by our clinical cases. Usually, organ/vascular dissections are the presenting signs in most adults with vEDS. In some cases, arterial rupture may be preceded by arteriovenous fistulae, aneurysms, or dissection but it may also occur spontaneously. Most subjects with vEDS (60%), who are diagnosed before 18 years old, are identified because of a positive family history. In our patients’ situation, due to circumstantial factors, the family link could not be established. When family inheritance is suspected, specific clinical exams and tests should be performed on neonates as soon as possible, especially when identifying specific morphological and clinical signs.

To better understand the evolution of patients with vEDS, particularly these two clinical cases, we should look back to vEDS from a holistic point of view. The most comprehensive descriptions of clinical features and natural history of vEDS individuals arise from two types of studies: a cross-sectional and retrospective view obtained at the time of diagnostic testing and a nearly 15-year-long cohort study from one group in France (13, 15). A retrospective review of the health history of more than 1,200 individuals with vEDS outlined the natural course of the disorder (13). Most individuals were discovered due to a major complication (70%), at an average age of 30 years. Mean survival in the population was 50 years, with a younger mean survival in males (by 5 years) than in females, partially due to a higher rate of lethal vascular events in males than females before 20 years of age. Similar results were reported in the French cohort of 215 individuals with vEDS, but in this case, a difference in mean survival based on sex was not observed (15).

In our patients’ cases, unsuspected young adult patients present to the ER having a vascular potentially life-threatening event (vascular dissection or arterial occlusion), and only careful step by step approach and interdisciplinary clinical management led to correct diagnosis and treatment.

In the first patient we identified a carotid-cavernous fistula (CCF). CCF is an abnormal communication between the cavernous sinus venosus and the carotid artery, and can appear spontaneously or after a head trauma (3).

Barrow et al. proposed in 1985 a classification of carotid-cavernous fistulas into 4 types (4):

Type A: carotid-cavernous fistulas with full channel, high flow (5). Type A fistula is a direct, high-flow fistula between the internal carotid artery and the cavernous sinus. It is the most common type of CCF after head trauma, but it can also occur spontaneously in a predisposing vascular area such as in Ehlers–Danlos syndrome. The etiopathogenic hypothesis of direct fistulas assumes the formation from a traumatic rupture of the wall of the cavernous internal carotid artery or because of the rupture of an aneurysm. Thus, arterial blood with high pressure gains rapid access to the venous system and leads to venous hypertension (6).

Type B: dural fistulas with low flow, fed by the branches of the intra-cavernous internal carotid artery.

Type C: dural fistulas with reduced flow, fed by the cavernous branches of the external carotid.

Type D: low-flow dural fistulas supplied by both internal and external carotid branches (5).

BD types, or indirect fistulas, occur between the meningeal branches of the external or internal carotid artery and the cavernous sinus (7). These are low-flow fistulas. Symptoms are usually milder and may include dilatation of conjunctival and episcleral vessels and proptosis, diplopia (6).

Once the diagnosis of CCF is evoked, it is possible to visualize the carotid-cavernous fistula on dynamic angio-MRI or through an arteriography which remains the reference examination for diagnosis and which makes it possible to plan the therapeutic management (8).

This rare pathology is a therapeutic emergency because it involves a vital and visual prognosis and requires a close collaboration between radiologists, neurosurgeons, and ophthalmologists. Current treatment of CCF is based on interventional neuroradiology (9). Its principle is the occlusion of the fistula by inserting removable intravascular balloons or metal coils, as was the case with our patient, while respecting the patency of the carotid axis. The approach can be done *via* the arterial route (inferior and posterior fistula) or retrograde venous route (superior and anterior fistula) (12). Neurosurgical treatment by arterial ligation retains its place in case of failure of endovascular therapy (5).

In the second patient’s case, the presentation diagnosis was CRAO. CRAO is not a frequent diagnosis in a young person; when such an event occurs, it is very important to establish what caused it, since it is uncommon at such young age. In this situation, properly investigating the patient and diagnosing the vascular dissections saved patient’s life and further led to the underlying cause. At this moment, the patient is aware of his condition, takes proper anti-coagulant medications, avoids any intense physical efforts, and has a good life quality.

Diagnosing vEDS is not an easy task. The genetic tests establish the certainty diagnosis, but sometimes it may become difficult for a patient to acquire these tests, usually for objective reasons. As previously pointed out, diagnosis of vEDS is established in a proband by identification of a heterozygous pathogenic variant in *COL3A1*, or, when molecular genetic testing does not identify a *COL3A1* pathogenic variant, on biochemical analysis of type III procollagen from cultured fibroblasts, or even by histopathological exam of biopsy tissue, which could be difficult but orients the diagnosis in the right track. In the first case, the female patient, the diagnosis was made by genetic analysis, in the latter, the male patient, the strong(er) suspicion was made after the skin biopsy and genetic tests confirmed the suspicion.

Further management of the vEDS cases include proper and prompt treatment of manifestations. Patients with vEDS may face a lot of challenges and unexpected events. As such, affected individuals are instructed to pursue immediate medical attention for sudden, unexplained pain. Patients with vEDS require periodic arterial screening by ultrasound examination, magnetic resonance angiogram, or computed tomography angiogram with and without venous contrast. It is highly recommended blood pressure monitoring on a regular basis, to be able to identify as early as possible any sign of hypertension.

All the manoeuvers that may cause vascular injury to the patient should be carefully considered. Arteriography should be discouraged and used only to identify life-threatening sources of bleeding prior to surgical intervention; routine colonoscopy in the absence of concerning symptoms or a strong family history of colon cancer should be avoided; elective surgery should be considered only if the benefit for the patient is expected to be substantial.

Patients should be instructed to avoid any kind of trauma, including collision sports or weight training with extreme lifting.

When having a female young patient diagnosed with vEDS, it is important to advise the patient regarding pregnancy risk. It is known that affected women have a 5% mortality risk with each pregnancy. Thus, female patients should be informed about the maternal risks and, if pregnant, should receive a careful follow up in a high-risk obstetric program.

Genetic counseling is another issue to be considered when referring to vEDS patients. vEDS is frequently inherited as an autosomal dominant disease, but on some occasions it may have a biallelic inheritance. That means that 50% of affected individuals inherit the *COL3A1* pathogenic variant from an affected parent, and about 50% of affected individuals have a de novo pathogenic variant. Each child of an affected individual has a 50% chance of inheriting the pathogenic variant and developing the disorder. Prenatal testing for a pregnancy at increased risk and preimplantation genetic testing are possible in families in which the pathogenic variant in *COL3A1* has been identified.

Last, but not least, vEDS patients should carry documentation of their genetic diagnosis, such as an emergency letter, MedicAlert®, or vEDS “passport” (4, 12).

Although rare, vEDS is a syndrome that one may encounter in an interdisciplinary emergency hospital (the two cases presented in the ER at 1 year distance); it is important to recognize the signs of such disease, since the right diagnosis, treatment, and prevention of further lesions, may make the difference between life and death. Each patient may have different clinical aspects that require different approaches, so being aware of all sides of this pathology and keeping an open mind as clinicians could make a difference in these cases, regardless of the gravity of their clinical state.

## Ethics statement

Written informed consent was obtained from the individual(s) for the publication of any potentially identifiable images or data included in this article No. 15003/13.03.2023.

## Author contributions

MC, RP, and DB contributed to the conception and design of the manuscript, the acquisition, analysis, and interpretation of data, the drafting of the work, revision and editing all data to cover all aspects for the proper intellectual content. RI contributed to the acquisition, the analysis and interpretation of data of the study, to the drafting of the work and its critical revision for important intellectual content. GG and AC contributed to the conception and design of the manuscript. MC contributed to the drafting of the work and its critical revision for important intellectual content. MR contributed to the drafting of the work and its critical revision for important intellectual content. MZ, GI, and VV contributed to the drafting of the work. AS contributed to the acquisition and interpretation of data, and final revision of the manuscript. All authors read and approved the final version of the manuscript and agreed to be accountable for all aspects presented in the paper in ensuring that questions related to the accuracy or integrity of any part of the work are appropriately investigated and resolved.

## Conflict of interest

The authors declare that the research was conducted in the absence of any commercial or financial relationships that could be construed as a potential conflict of interest.

## Publisher’s note

All claims expressed in this article are solely those of the authors and do not necessarily represent those of their affiliated organizations, or those of the publisher, the editors and the reviewers. Any product that may be evaluated in this article, or claim that may be made by its manufacturer, is not guaranteed or endorsed by the publisher.
